# A Case study of the radiation therapy treatment of a transitional cell carcinoma of the distal urethra

**DOI:** 10.1002/jmrs.371

**Published:** 2020-01-16

**Authors:** Amy Donovan, Rachael Beldham-Collins, Sandra Turner

**Affiliations:** ^1^ Crown Princess Mary Cancer Care Centre Westmead Hospital Westmead NSW Australia

**Keywords:** Case study, radiation therapy, radiation oncology, urethral carcinoma, Transitional cell carcinoma

## Abstract

Urethral carcinoma is a rare urological cancer, accounting for only 1% of malignancies in Australia. The most common histology is transitional cell carcinoma (TCC). The majority of these cancers are treated with surgery. The main purpose of this case study is to describe a novel radiation treatment technique for treatment of this uncommon cancer. This report details organ‐preserving treatment for a distal penile urethral cancer using definitive radiation therapy (RT). In May 2016 a 69‐year‐old male presented to Crown Princess Mary Cancer Centre (CPMCC) with a small TCC of the distal urethra. The patient was offered numerous treatment options, both radical and organ‐preserving approaches, and came to a final decision of a course of radiation therapy despite the lack of randomised evidence to guide treatment in this setting. A dose of 66 Gy in 33 fractions from parallel opposed lateral beams was prescribed to the distal penile urethra. This case required an unusual approach to patient set up to allow access for accurate treatment delivery and to maintain patient comfort. The patient tolerated the full course of radiation therapy with expected skin side effects. He has maintained adequate penile function and is currently free from disease at 33 months with ongoing clinical follow‐up.

## Graphical abstract

A case study on a distal urethral TCC treated with radiation therapy at the Crown Princess Mary Cancer Centre. The purpose of this case study is to describe a novel radiation treatment technique for a rare site as the patient refused typical treatment regimes due to quality of life implications. The patient tolerated a full 66Gy in 33 fractions radiation therapy course and has since returned to his normal lifestyle cancer‐free.

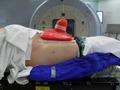

## Introduction

Primary urethral carcinomas are very rare, constituting 1% of all malignancies diagnosed each year in Australia.^1^ Urethral carcinomas in the 75‐84 year age group are approximately four times more common in men than women. The age‐standardised rate for men in this age group is approximately 4.3 people/million compared to women at 1.5 people/million. This diagnosis is even more rare in patients less than 55 years of age (incidence of 0.2 per million).^2^ The much longer male urethra compared to the female urethra (15‐20 cm in length compared to 4 cm) likely accounts for the gender differences in incidence. The male urethra is divided into five main sections or structures including the bulbous, pendulous, fossa navicularis, membranous and prostatic urethra. More commonly it is called either the proximal or distal urethra.^3^

Urethral carcinomas histopathologically fall into three main morphologies: transitional cell carcinoma (TCC), squamous cell carcinoma (SCC) or adenocarcinoma (AC) in order of prevalence. TCCs comprise 54‐62% diagnoses, followed by SCC (16‐22%) and AC (10‐16%).^1^, ^4^

TCCs arise from the transitional epithelial layer of the urinary tract. They are most commonly found in the bladder or the upper urinary tract. It is incredibly rare to have a primary distal urethral carcinoma, resulting in a lack of evidence to guide modern treatment approaches and no standardised treatment protocols worldwide.^5^, ^6^ Traditionally, distal urethral carcinomas were treated with a partial or complete penectomy and have a better prognosis.^1^, ^6^.

There were very few studies that investigated radiation therapy as a sole treatment option for distal urethral TCCs. Janisch et al., 2019, state that radiation therapy alone has a limited role in genital preserving treatment due to its decreased survival and recurrence rates.^9^ All studies completed evaluating the treatment regimes for penile carcinoma to date have very small sample sizes which makes reliability questionable, and majority of the information available is outdated.^7^, ^8^. The purpose of this paper is to review our experience of treating a distal urethral TCC using external beam radiation therapy over other treatment options to inform the wider radiation therapy community.

## Case Presentation

In 2016, a 69‐year‐old male patient was referred by a urologist for a discussion about management options. He had an Eastern Cooperative Oncology Group performance status (ECOG) of 0, was an ex‐smoker and had no significant comorbidities. The patient was initially diagnosed in 1999 with a very small patch of SCC of the glans penis after presenting with penile bleeding. No further tumour cells were evident on second biopsy, presumably the tiny lesion being completely excised by the initial biopsy. The patient remained symptom‐free and under observation. In 2016, a new nodule measuring a few millimetres appeared just inside the external urethral meatus. Repeat biopsies at this time revealed a primary invasive distal urethral transitional cell carcinoma (TCC) localised in the distal 1.5 cm of the penile urethra with no nodal involvement. No further clinical staging investigations were undertaken.

Radical surgery had been recommended as the ‘gold standard’ based on the limited available literature. A penectomy was recommended with urethral diversion to the perineum. Alternatively, penile preserving surgery or radiation therapy was offered. Both surgical options would impact the patient's sexual activity and were considered unsuitable by the patient.

The Radiation Oncologist (RO) further discussed with the patient and his wife the lack of research into the effectiveness of radiation therapy alone for his tumour type and location. The patient was informed of the potential risks including reduced prognosis skin irritation, dysuria, pain and also late potential side effects of stricture.^9^ The patient consented to undergo a course of radiation therapy due to the previously disclosed psychological reasons. The RO prescribed 66 Gy in 33 fractions, 10 fractions per fortnight.

## Discussion

### Simulation

Radiation Therapists brainstormed appropriate set up positions prior to simulation to take into account, comfort, stability, reproducibility and patient dignity. The patient remained comfortable and compliant during the simulation appointment which enabled a suitable set up to be achieved.

**Figure 1:**
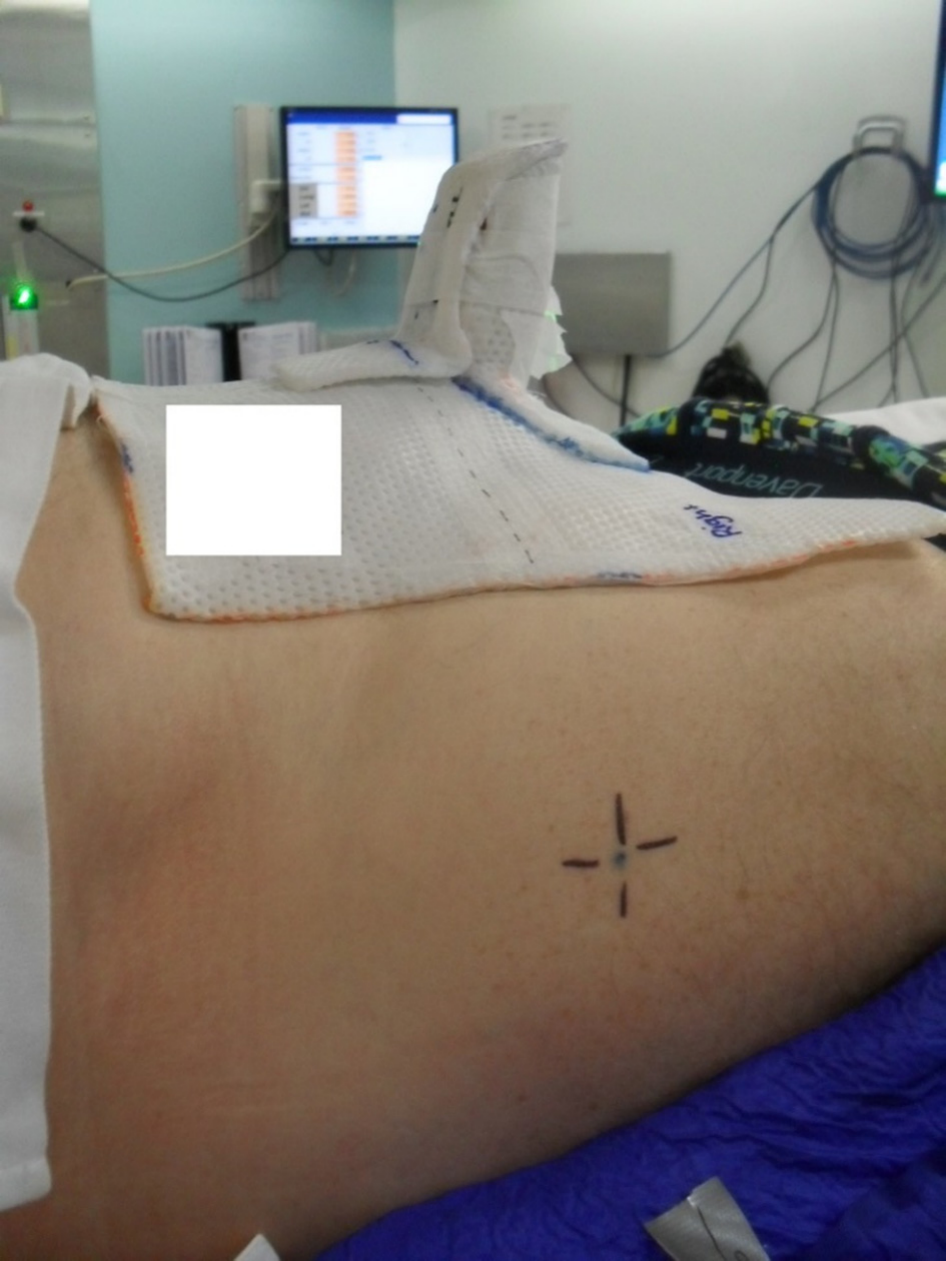
Customised or fit support and tattoo location.

**Figure 2:**
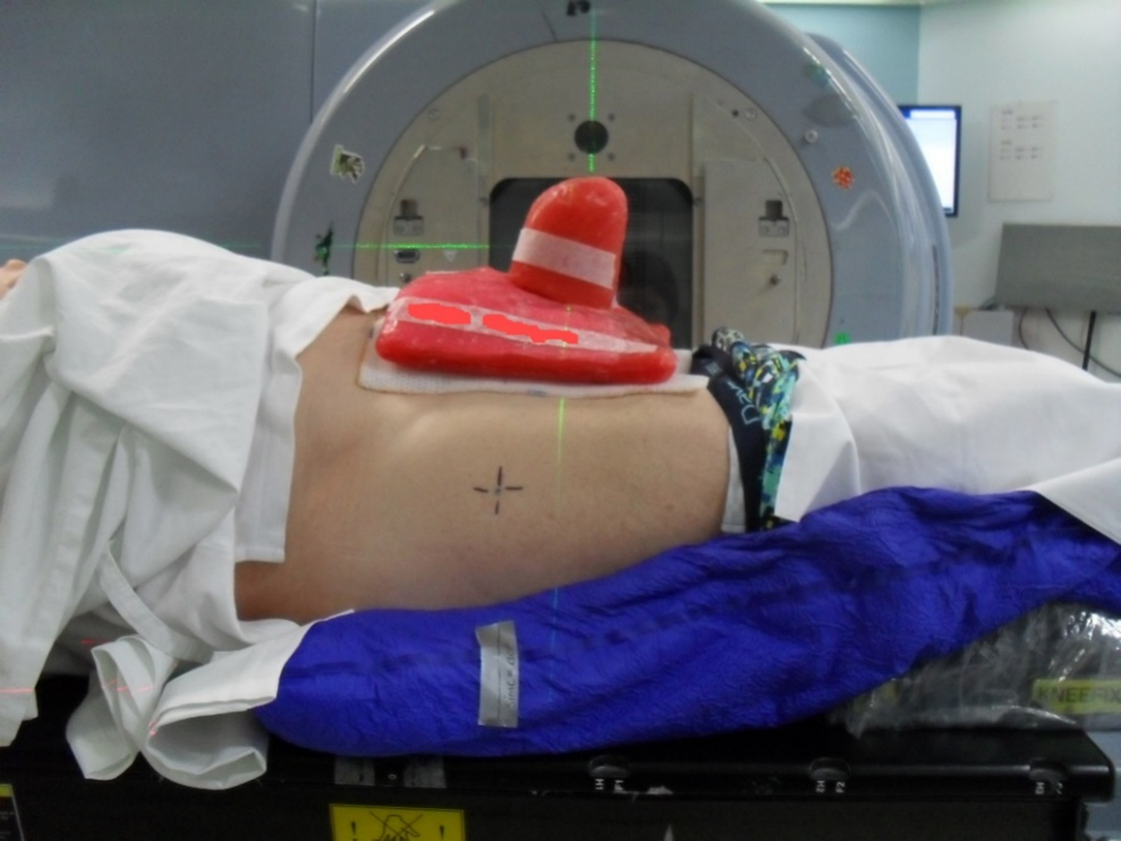
Personalised wax block and vacbag created in simulation.

Set up involved a vacbag being formed under the patient's lower body and over the top of an indexed black kneelok to maintain stability. The patient's penis was tied distally with gauze to hold the organ abducted and perpendicular to the patient's body. A thermoplastic stand was created to support the inferior aspect of the penis, which the gauze was then pulled over the top of this thermoplastic stand and taped down to hold the penis in place for the duration of treatment. A thermoplastic cover was made to support the superior aspect of the penis. Once all aspects of the support mechanisms were created, the anatomical base of penis (BOP) was chosen as the simulation centre, and two lateral tattoos were placed for levelling purposes. Reference marks were placed on the vacbag as well as the thermoplastic supports to align with BOP and the lateral tattoos to assist with daily reproducibility. (Figs. 1 and 2).

The simulation took approximately 1.5 hours which was significantly longer than originally anticipated; however, the patient remained relaxed throughout the procedure.

During the moulding of the thermoplastic support in simulation, temperature created a number of set up issues. The thermoplastic was initially warm, causing the organ to swell. As the thermoplastic cooled down, the swelling decreased which caused gaps between the thermoplastic and the penis. For treatment, it was decided to pack these gaps with wet cotton wool to reduce scatter of the radiation beam.

The computerised tomography (CT) scan was performed with the thermoplastic support in place. A wide bore Lightspeed GE CT scanner with a scan slice thickness of 0.25 cm was used for the procedure. A personalised wax block was created as per the Radiation Oncologists prescription. Simulation and mould room staff created the personalised wax block using numerous layers of wax sheets and the thermoplastic support as a base.

Once the 2 cm uniform thickness had been reached, a CT scan was performed on the wax block to confirm thickness and ensure no air bubbles were present.

This process took a few days to complete.

### Planning

**Figure 3:**
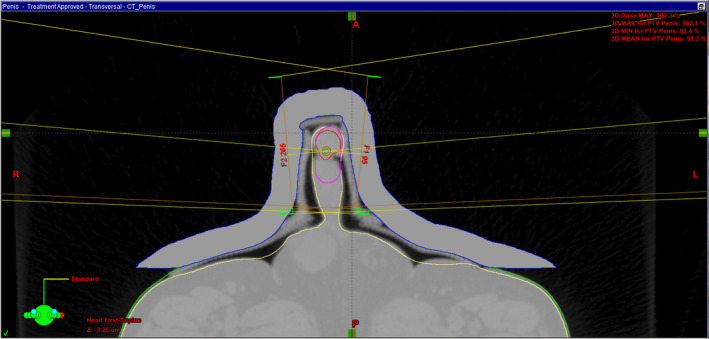
The chosen beam angles, wax bolus placement in blue, GTV in red and CTV in pink.

The 3DCRT beam arrangement used two lateral 6MV photon beams planned using the Varian Eclipse planning software version 13.7.. The beams were angled to reduce dose to the patient's scrotum. 6MV was the chosen energy due to the small separation. (Fig. 3) The 2 cm wax block was generated on the eclipse planning system and given a density of 1 to save the patient returning for a second CT scan. The bolus effect of the wax block ensured the penis was receiving maximum dose.

The Radiation Oncologist contoured a gross tumour volume (GTV) to the tip of the penis, a clincal target volume (CTV) which included an extra 2 cm of the penile shaft and then a 1 cm margin around this to create the planning target volume (PTV) to ensure sufficient coverage of the tumour. The plan conformed to the ICRU 83 guidelines with the PTV receiving 98% of the dose and a point maximum dose of 102.3% which was located in the bolus. Organ at risk (OAR) doses met constraints with the scrotum receiving less than 4 Gy, and no dose being recorded for the bladder and heads of femur.

### Treatment issues

Treatment started 16 working days after simulation. The patient was treated on a Varian Clinac 21iX machine. Fraction 1 proved the set up to be reproducible with all tattoos lining up with the vacbag and the thermoplastic fitting appropriately. The patient was compliant and in a state of repose during his first treatment indicating his level of comfort with the staff and the process.

**Figure 4:**
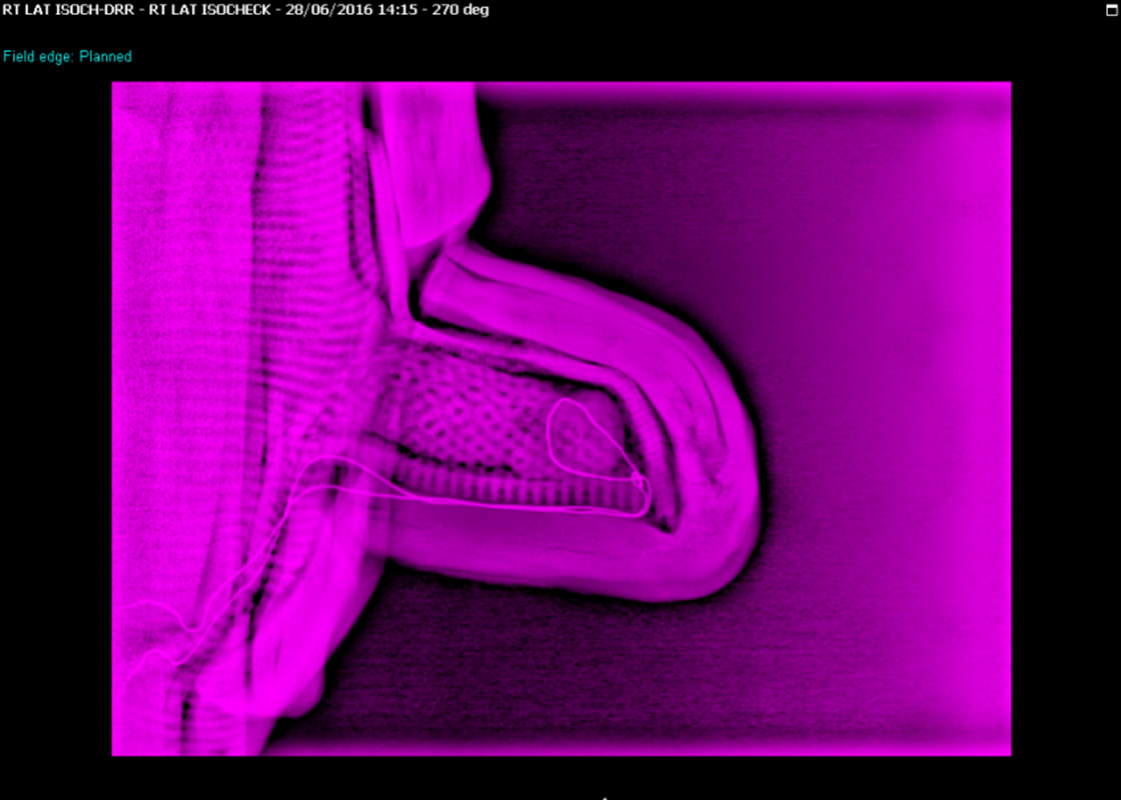
The treatment verification image obtained on day 1 of radiation therapy.

The first fraction required treatment staff determine the most suitable kilovoltage (kV) filter for the treatment verification images prior to irradiation in order to provide an accurate delineation of the penile soft tissue to verify simulation set up was replicated, and acceptable bolus placement was achieved. Most predefined kV filters only provided adequate visualisation of bony anatomy, which given the need for precise soft tissue delineation, rendered these inappropriate for use in this case. Staff settled on the use of the ‘Extermity’ filter (Kv 65 mAs 3.5) as it displayed the best soft tissue visualisation to enable accurate set up confirmation. (Fig. 4).

**Figure 5:**
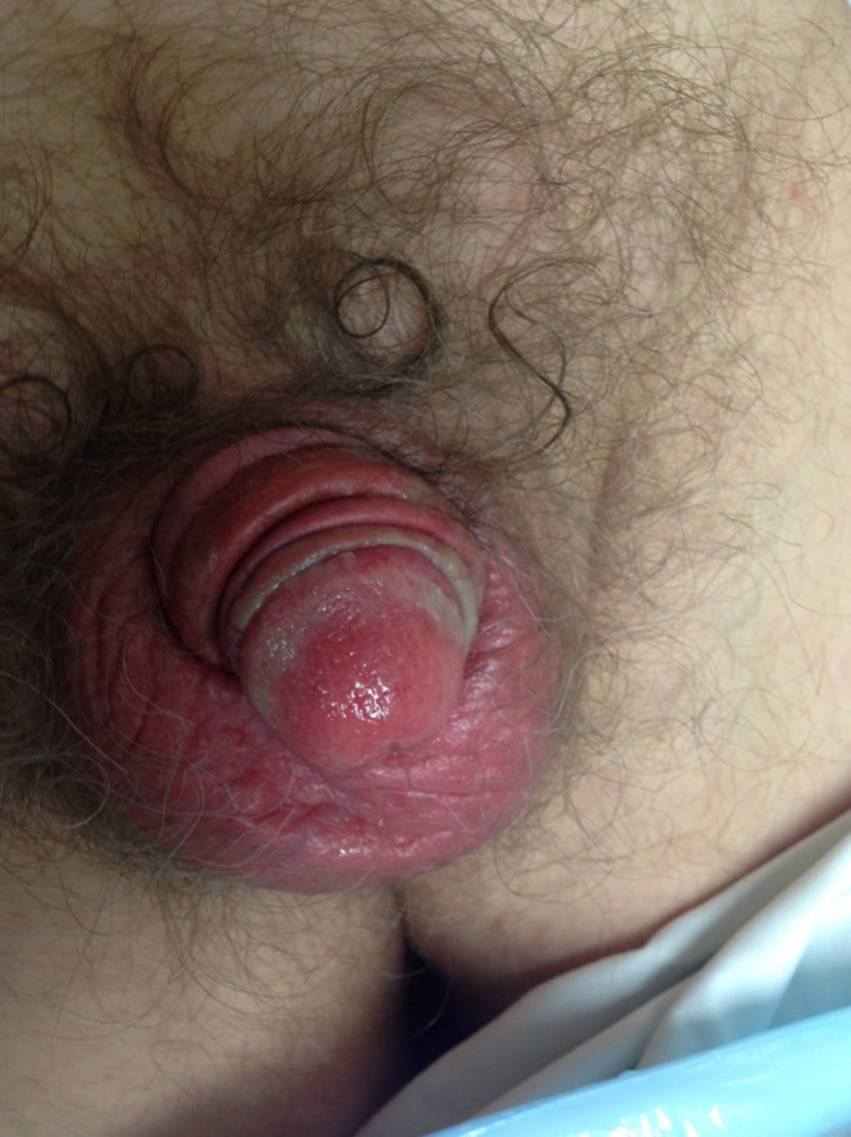
The patient experience pain, swelling and a severe skin reaction visible here.

The final issue we faced during the patients treatment journey was acute side effects including swelling and pain. The swelling of the penis made it difficult to position it through the thermoplastic support on a daily basis. (Fig. 5) In the final week of treatment, some of the thermoplastic needed to be cut away due to this swelling of the organ and to allow treatment staff to reproduce the same treatment position; however, this did not alter the plan in any way.

It also meant that wet combine packing was no longer required for the procedure. The pain the patient was experiencing also made it difficult to tie gauze around the penis daily. The patient was prescribed paracetamol to help relieve this side effect.

The patient suffered from moist desquamation to the penile shaft and glans while his scrotum experienced swelling and erythema. Despite the acute side effects experienced, the patient continued to urinate normally for the entire treatment, aided by the use of a dilator provided by the oncology team.

### Follow‐up

**Figure 6:**
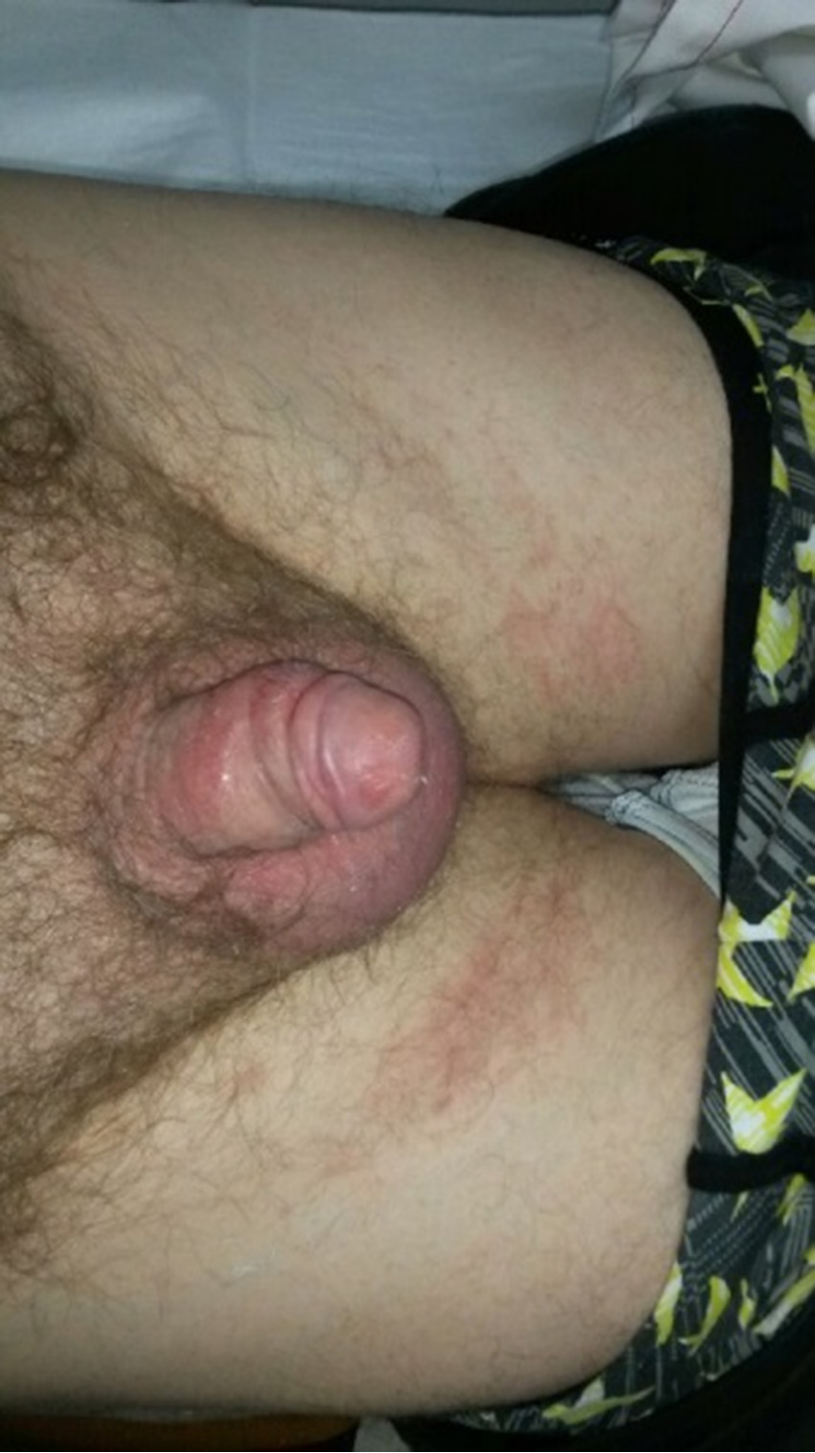
Skin reaction 8 weeks post‐treatment.

After completion of radiation therapy, the patient attended for weekly skin review and dressings until the acute side effects subsided. The patient was treated with daily Solugel^TM^ and Jelonet^™^ dressings to the penis and sorbolene cream to the scrotum. During the initial weekly reviews, skin reaction to the penile shaft and glans worsened; however, the scrotal reaction also increased. He developed pruritus (itchiness) to his scrotum and eventually his upper thighs. He also experienced slight dysuria (painful urination) during the first few weeks of his follow‐up. At 3 weeks post treatment, it could be seen that the moist desquamation of the penile shaft was subsiding and the swelling of the scrotum had reduced; however, pruritus of the scrotum was worsening. During a visit to the department, at 8 weeks post‐treatment, it was noted that the rash returned to the patient's upper thighs as well as scrotal oedema. (Fig. 6) The patient was diagnosed with an allergic reaction to sorbolene cream in the scrotal and thigh region. This area was then treated as a fungal infection using Hydrozole^™^ cream bi daily for a week, along with Calmaseptine^™^ cream to the penile shaft; his symptoms quickly improved. After a few more weeks of the daily cream regime, the skin infections cleared. At 10 weeks post‐treatment, the patient regained normal urinary function with good stream and denied any late side effects including dysuria, haematuria or sensation loss. His energy levels had returned to a normal baseline, and he was maintaining normal sexual function.

In February 2017, 6 months post‐treatment, the patient presented to the clinic fit and well. On examination, it was noted the patient had telangiectasia (spider veins) over the penile shaft but otherwise his skin had returned to normal colour. Upon multidisciplinary discussions between the oncologist and urologist, it was agreed upon that because there is no visible abnormality, the patient did not require to be sent for further biopsies but proceeded to follow a 6 monthly specialist review plan.

At the 12‐month, 18‐month, 24‐month and 30‐month reviews, it was noted that some mild dryness and oedema over the glands of the penis was observed, telangiectasia still present; otherwise, the area appeared normal. No lymphadenopathy or other evidence of disease was visible. There was no change in urinary or sexual functions. At 24 months post‐treatment, he was still using daily moisturising cream to aid in dry skin control which he continues to do so to date. He required self‐catheterisation to urinate however managed this well on his own at home. The patient was training for the trek to base camp in Nepal, which he has since successfully completed.

The patient is now 33 months post treatment and shows no sign of recurrence. In this instance, treatment with radiation therapy has demonstrated similar local control to that reported by Smith et al. who discussed penile preserving surgery for patients with distal urethral tumours.^7^ Similarly, the local control presented in this case study is comparable to that achieved by Gheiler et al. reporting on two cases of distal urethral cancer treated with partial or complete surgery and adjuvant chemotherapy and radiation therapy.^8^

## Conclusion

A standardised treatment regime for TCCs of the distal urethra has not been established due to low incidence rates. Whilst radiation therapy has rarely been utilised for this diagnosis, the patient discussed in this case report found the treatment process and overall outcome to date to be satisfactory with tumour control achieved. Continued follow‐up of 5 years is required for a definitive outcome to be recorded.
